# Early Detection of MCI based on both EEG and NIH‐Toolbox Cognition Scores

**DOI:** 10.1002/alz70856_104092

**Published:** 2025-12-24

**Authors:** Ming Gu, Boxin Sun, Voyko Kavcic, Jian Ren, Bruno Giordani, Tongtong Li

**Affiliations:** ^1^ Michigan Alzheimer's Disease Research Center, Ann Arbor, MI, USA; ^2^ Michigan State University, East Lansing, MI, USA; ^3^ Wayne State University, Detroit, MI, USA; ^4^ International Institute of Applied Gerontology, Ljubljana, Slovenia; ^5^ University of Michigan, Ann Arbor, MI, USA

## Abstract

**Background:**

Early identification of those at risk of developing Alzheimer's disease or a related dementia plays a determinant role for the implementation of timely interventions and improving quality of life. This study investigated whether the classification accuracy of normal cognition (NC) and mild cognitive impairment (MCI) can be improved by adding NIH Toolbox‐Cognition Battery (NIHTB‐CB) data to the EEG‐based analyses.

**Method:**

In our sample set, there were 71 participants (*n* = 71, 40 NC and 31 MCI) who had both the EEG (64‐channel) and NIHTB‐CB data, acquired at Wayne State University and Michigan Alzheimer's Disease Research Center. The participants received two (eye‐closed) resting state electroencephalograms and between which they engaged in a visual motion direction discrimination task. The NIHTB‐CB included tests of Crystalized ability—Oral Reading Recognition and test of Picture Vocabulary, and measures of Fluid ability— Dimensional Change Card Sort (DCCS), Pattern Comparison for processing speed, Flanker (inhibition and attention), List Sorting (working memory), and Picture Sequence Memory.

We explored three approaches: EEG alone, NIHTB‐CB alone, and EEG plus NIHTB‐CB, and compared their performance for k‐fold (k = 3, 5, 10) test accuracy. Here the results were averaged over 50 rounds, and in each round, the test data (1/k of the sample set) was selected randomly and the rest of the data ((k‐1)/k portion of the sample set) was used to train the discrimination model.

**Result:**

The 10‐fold test accuracy is 75.49% for the EEG model, 65.89% for the NIHTB‐CB model, and 78.82% for EEG plus NIHTB‐CB model. The 10‐fold cross validation accuracy (i.e., where all the samples were used for feature selection) is 93.1% for EEG plus NIHTB‐CB model. Similar trend was observed for k = 3 and 5.

**Conclusion:**

Our results indicated that for MCI detection, the EEG model is more sensitive than the NIHTB‐CB model, but the EEG plus NIHTB‐CB model delivers the best results, which implies that cognitive assessment scores could help improve the discrimination accuracy of the EEG model. Our study also showed that feature selection and classification become more reliable as the sample size increases.

**Funding**: NSF‐2032709/Li; NIH‐1R21AG046637‐01A1/Kavcic and NIH‐1R01AG054484‐01A1/Kavcic; NIH‐P30AG072931/Paulson; NIH‐P30AG024824/Yung.

